# EEG time–frequency analysis reveals blunted tendency to approach and increased processing of unpleasant stimuli in dysphoria

**DOI:** 10.1038/s41598-022-12263-9

**Published:** 2022-05-17

**Authors:** Carola Dell’Acqua, Elisa Dal Bò, Tania Moretta, Daniela Palomba, Simone Messerotti Benvenuti

**Affiliations:** 1 Department of General Psychology, University of Padua, Via Venezia 8, 35131 Padua, Italy; 2 Padova Neuroscience Center (PNC), University of Padua, Via Orus 2/B, 35131 Padua, Italy

**Keywords:** Emotion, Motivation, Human behaviour, Depression

## Abstract

To date, affective and cognitive processing of emotional information in individuals with depressive symptoms have been examined through peripheral psychophysiological measures, event-related potentials, and time–frequency analysis of oscillatory activity. However, electrocortical correlates of emotional and cognitive processing of affective content in depression have not been fully understood. Time–frequency analysis of electroencephalographic activity allows disentangling the brain's parallel processing of information. The present study employed a time–frequency approach to simultaneously examine affective disposition and cognitive processing during the viewing of emotional stimuli in dysphoria. Time–frequency event-related changes were examined during the viewing of pleasant, neutral and unpleasant pictures in 24 individuals with dysphoria and 24 controls. Affective disposition was indexed by delta and alpha power, while theta power was employed as a correlate of cognitive elaboration of the stimuli. Cluster-based statistics revealed a centro-parietal reduction in delta power for pleasant stimuli in individuals with dysphoria relative to controls. Also, dysphoria was characterized by an early fronto-central increase in theta power for unpleasant stimuli relative to neutral and pleasant ones. Comparatively, controls were characterized by a late fronto-central and occipital reduction in theta power for unpleasant stimuli relative to neutral and pleasant. The present study granted novel insights on the interrelated facets of affective elaboration in dysphoria, mainly characterized by a hypoactivation of the approach-related motivational system and a sustained facilitated cognitive processing of unpleasant stimuli.

## Introduction

Ranked among the world’s most common and economically burdensome conditions, depression is a mood disorder that results in sustained negative affect and/or loss of interest in pleasant activities^1,2^. Dysphoria is a condition characterized by depressive symptoms that does not meet the criteria for a formal diagnosis of major depression with respect to the frequency, duration and/or severity of symptoms^3^. Dysphoria is an acknowledged risk factor for the development of clinical depression measured at follow-up assessments of up to 4 years (e.g.^4,5^). Studying dysphoria has several advantages, as it represents a risk condition for the onset of clinical depression, and it allows analyzing early depressive symptoms without any confounds provoked by the chronicity of the disorder or by the intake of antidepressant medications.

A core feature of depressive symptoms is dysregulated affective disposition^6^. Particularly, symptoms of sadness and distress have been linked to the activation of the withdrawal-related motivational system, which is primarily activated in contexts of threat^7^. Conversely, symptoms of anhedonia, psychomotor retardation and apathy are linked to the hypoactivation of the approach-related motivational system^8^. Dysregulation of affective disposition can be assessed by examining emotional responding, namely a multifaceted affective process involving subjective, behavioral, and physiological adjustments to affective experience^9^. Of note, emotional responding has been widely studied in relation to the development and maintenance of depressive symptoms and three main hypotheses have been put forward (e.g.^10^). Firstly, the negative potentiation hypothesis holds that negative mood tends to potentiate emotional responding to unpleasant stimuli, indicating a heightened activation of the withdrawal-related motivation system (e.g.^11,12^). Although this model is coherent with depression’s feature of sustained negative affect, it is not fully supported by recent empirical evidence suggesting, instead, that depressed mood is mostly linked to a reduced emotional responding to positively valenced or rewarding stimuli (e.g.^10,13–19^). This second view, known as the positive attenuation hypothesis, holds that depressive symptoms are mostly linked to reduced emotional responsiveness to pleasant content, indicating a hypoactivation of the approach-related motivational system in the brain^14,15,17–20^. Notably, the hypoactivation of approach-related motivation represents an important risk factor for the development of depression^8,21^. Moreover, within the Research Domain Criteria (RDoC matrix^22^), is included a dimension believed to be a potentially unique feature contributing to depression, namely the hypoactivation of the Positive Valence System^23^. The positive attenuation hypothesis has been extended to a third alternative, the emotional context insensitivity (ECI) hypothesis^10^, which holds that depression is characterized by a hypoactivation of both motivational system^10,24–26^. Meta-analytic evidence provided support for the positive attenuation and the ECI model, but not for the negative potentiation hypothesis^20,25^.

In addition to dysregulated affective disposition, altered cognitive processes of affective content have been shown to play a critical role in the development and maintenance of depressive symptoms. Particularly, according to classical cognitive models of depression, negative self-referential schemata, characteristic of depression, affect cognitive processing and, particularly, attention^27^. The facilitated processing of negative information is believed to contribute to the etiopathogenesis and maintenance of depressive symptoms^28–30^. Compared to controls, individuals with depressed mood showed increased orienting and processing of negatively valenced stimuli (e.g.^31–34^; for a review see^29^). This mechanism has been suggested to generate a rigid pattern of negative appraisal of unpleasant events, which results in an increased difficulty to reappraise and regulate emotions^31,35^. Further, depression appears also to be characterized by reduced processing of pleasant stimuli (e.g.^33,36^; for a review see^37^), indicating attentional avoidance of pleasant content.

To date, affective disposition and cognitive processing of affective content, two synchronous mechanisms, have been jointly examined through peripheral psychophysiological measures or event-related potentials (ERPs) (e.g.^7,38,39^). Nevertheless, an advantageous measure that can be employed is the time–frequency analysis of electroencephalographic (EEG) activity within specific frequency bands while participants are exposed to affective vs. neutral content. Indeed, time–frequency analysis allows the extrapolation of information that is not available using ERPs analysis and reflects distinctive aspects of information processing (e.g.^40^). Specifically, affective disposition can be assessed by analyzing delta (1–3 Hz) and alpha (8–12 Hz) frequency bands. Although delta oscillations are considered a correlate of cortical inactivation prominent during sleep^41^, recent studies have demonstrated that delta rhythm across spatially distributed cortical regions sustains basic motivational drives, especially towards pleasant and rewarding stimuli^42^. Indeed, delta oscillations appear to have a functional role in monitoring the motivational relevance of affective cues and in the identification of pleasant/rewarding stimuli and are generated by subcortical regions involved in the motivational system^42–45^. Studies have shown that event-related delta power is increased by emotionally salient cues (unpleasant and pleasant) as compared to neutral ones mostly in centro-parietal regions^42,46–50^. However, to date, delta power in individuals with depressive symptoms during a picture viewing task has not been fully explored. Furthermore, alpha band, a measure considered to be inversely related to the level of cortical activation^51^, is thought to be an indicator of affective disposition^52^. An asymmetric pattern of alpha activity, with increased alpha in the left frontal lobe compared to the right, reflects a hypoactivation of the approach-related motivation system and has long been considered to represent a potential biomarker for depression in resting-state conditions (^52,53^, for a review see^54^) and even more in emotional contexts^55^. However, to date, only a few studies have examined alpha asymmetry during emotional processing in dysphoria or depression^15,18,56,57^. Also, most studies have analyzed alpha activity only at anterior scalp sites, even if asymmetry in the alpha in depression has also been reported at posterior scalp sites. Indeed, individuals with or without familiarity for depression showed a right temporo-parietal dysfunction, as indexed by higher alpha activity^18,56,58^. Particularly, a smaller alpha desynchronization (i.e., higher alpha) in frontal and right centro-parietal regions to pleasant images was found in dysphoria^18^. Given that right parietal activity is thought to reflect arousal^56,58^, these results were interpreted as an under-engagement of the approach-related motivational system in individuals with dysphoria.

Furthermore, theta band (4–8 Hz) reflects the processing of salient events and can be employed to assess cognitive processing during the viewing of affective content^59^. Specifically, theta, distributed within a large network of brain regions involved in multimodal sensory and cognitive processing (^60,61^; for reviews see^62,63^), is believed to have a role in orienting and processing of arousing stimuli^62,64^. Congruently, theta oscillations are prevalent in superficial cortical layers in a widespread distributed fashion, supporting its role in the optimization of perceptual features in the environment (e.g.^65^). Moreover, considering that theta connections encompass subcortical limbic structures, theta activity could embody corticolimbic pathways involved in the cognitive integration of emotional information^66^. As a matter of fact, a greater event-related theta power for affective vs. neutral pictures at bilateral fronto-posterior sites was reported (e.g.^67,68^) and was suggested to reflect the role in the integration of affective and cognitive aspects of attentional operations^43,49^. To date, only a few studies have examined theta during affective processing in individuals with depression or dysphoria. An early frontal (~ 200–250 ms) weaker theta in response to vocalized emotional cues was reported in individuals with depressed mood^69^. Despite emotional categories were analyzed as a unitary category, this pattern was interpreted as a deficit in cognitive processing of all salient content^69^. Accordingly, depressive symptoms are believed to be associated with reduced orienting and salience detection^70^. Further, reduced theta oscillations to pleasant cues and enhanced theta to unpleasant cues relative to neutral ones in individuals with dysphoria was previously observed, suggesting a higher cognitive processing of unpleasant stimuli and reduced processing of pleasant ones in this group^71^.

The present study aimed to simultaneously examine affective disposition and cognitive processing in individuals with dysphoria through the analysis of the time–frequency changes within delta, theta, and alpha bands during the passive viewing of pictures from the International Affective Picture System (IAPS) library^72^. The formulated hypothesis was twofold and was based on the abovementioned functional correlates of delta, theta and alpha bands. First, regarding affective disposition, the dysphoria group was expected to show a hypoactivation of the approach-related motivational system and, as suggested by the ECI model, a potential hypoactivation of the withdrawal-related motivational system. Specifically, the group with dysphoria was expected to show a smaller increase in delta band activity in response to pleasant and unpleasant vs. neutral pictures across spatially distributed cortical regions relative to controls. Also, considering that reviewed evidence supporting the role of alpha as a measure of the approach-related motivational system, the dysphoria group was expected to show a smaller alpha desynchronization in the left frontal and right parietal cortex in response to pleasant (but not neutral and unpleasant) stimuli relative to controls. Second, regarding cognitive processing, a facilitated cognitive processing of unpleasant and a reduced processing of pleasant stimuli was expected in the group with dysphoria. Namely, these processing patterns would be indexed by increased theta activity to unpleasant relative to neutral stimuli and to controls and by a reduced theta activity to pleasant relative to neutral pictures and relative to controls.

## Methods

### Participants

The present study was conducted within an extensive research project on vulnerability to depression and participants’ EEG data have partially been described in a previous publication^33^. However, a distinct approach to data analysis was employed in the present study. A cohort of 85 Caucasian students at the University of Padua, Italy, voluntarily took part in the research project. The sample was medically healthy and free from psychotropic medication, as assessed with an ad-hoc anamnestic interview. In the present study, a group with dysphoria and a group without dysphoria were identified on specific criteria. Participants with dysphoria were identified by module A of the Structured Clinical Interview for DSM-5 (SCID 5-CV^73,74^) assessing current and past depressive symptoms. Furthermore, the Beck Depression Inventory-II (BDI-II^75,76^) was also employed for the assessment of depressive symptoms’ severity. Based on the psychological assessment, 27 participants (5 males) who scored equal to or greater than 12 on the BDI-II and showed at least two present depressive symptoms, for at least two weeks, without meeting the diagnostic criteria for major depression, persistent depressive disorder, or bipolar disorder, were assigned to the group with dysphoria. Twenty-five participants (12 males) who scored equal to or lower than 8 on the BDI-II and had no history of depression or current depressive symptoms were assigned to the control group (i.e., without dysphoria) (see^33^, for more details on participants enrolled in the study). To ensure separation between groups with dysphoria and without dysphoria, participants who scored between 9 and 11 on the BDI-II were excluded from the present study (*n* = 17). Also, individuals without depressive symptoms but with at least one past depressive episode (i.e., remitted, see^77^) were excluded from the present study (*n* = 16).

With respect to demographic variables, the two groups included in the analyses (with dysphoria, without dysphoria) did not differ in terms of age (*p* = .645; dysphoria group: Mean (M) = 20.7, standard deviation (SD) = 2.56, min = 18, max = 24; group without dysphoria: M = 20.4, SD = 1.72, min = 18, max = 28), sex (χ2 = 3.375, *p* = .066), and education (*p* = .920; dysphoria group: M = 15.0, SD = 1.56, min = 14, max = 18; group without dysphoria: M = 15.0, SD = 1.30, min = 14, max = 17).

Participants were given 13 € for their participation. All participants read, understood, and signed informed consent. The research was conducted in compliance with the World Medical Association Declaration of Helsinki on research on human subjects and was approved by the Ethical Committee of Psychological Research, Area 17, University of Padua (prot. no. 3612).

### Psychological measures

The Italian version of the mood episode module (module A) of the SCID-5-CV was employed as a reliable tool to assess the presence of dysphoria and to exclude individuals with major depression, persistent depressive disorder, or bipolar disorder. The SCID-5-CV was administered by a trained psychologist who had previous experience with administering structured clinical interviews. The Italian version of the BDI-II was also employed as a reliable measure of the severity of depressive symptoms in the past two weeks. The BDI-II is a self-report questionnaire composed of 21 items, each with a Likert scale of four-points and scores range from 0 to 63, where higher scores indicate greater depressive symptoms. In the Italian version, a score of 12 has been reported as the optimal cut-off score to discriminate between individuals with and without depressive symptoms^76^.

### Experimental task and procedure

Twenty-four pleasant (e.g., erotic couples, sports), 24 neutral (e.g., household objects, neutral faces), and 24 unpleasant (e.g., attacking humans and animals) color pictures (600 × 800 pixels) were presented to participants. Highly arousing pleasant and unpleasant pictures were selected from the International Affective Picture System (IAPS;^72^), since these have been observed to induce elevated psychophysiological changes (e.g.^7^). Pleasant and unpleasant pictures were matched for normative arousal ratings which were significantly higher than for neutral pictures. The numbers of the selected IAPS pictures are listed in the supplementary material.

Pictures were shown for 6000 ms each in a semi-randomized sequence (i.e., no more than one stimulus in the same emotional condition had to be shown consecutively). The length of picture presentation was set to 6000 ms as the project included the registration of electrocardiographic activity to analyze cardiac deceleration (as reported in^33^). Each picture was preceded by a 3000 ms interval where a white fixation cross was placed centrally on a grey screen. Participants were required to look at the central fixation cross and keep their gaze on the center of the screen. Picture presentation was followed by a variable intertrial interval (ITI) of 6000–8000 ms, during which a white fixation cross (identical to the 3-s baseline) was presented^33^.

Before the experimental session, participants were required to avoid alcohol consumption the day before and to avoid caffeine and nicotine the same day of the appointment. Upon arrival at the laboratory, after reading and signing written informed consent, participants were administered the ad-hoc anamnestic interview, the mood episode module (module A) of the SCID-5-CV, and the BDI-II. Then, participants were seated on a comfortable chair in a dimly lit, sound-attenuated room. After electrodes attachment and a 3-min resting-state period, six practice trials including two pleasant, two neutral, and two unpleasant pictures were provided. Then, participants underwent the emotional passive viewing task. The entire procedure took approximately 90 min.

### EEG recording

EEG data acquisition was accomplished using a computer running Eego software and using an Eego amplifier (ANT Neuro, Enschede, Netherlands). EEG was recorded using an elastic cap with 32 tin electrodes arranged according to the 10–20 System (Fp1, Fpz, Fp2, F7, F3, Fz, F4, F8, FC5, FC1, FC2, FC6, T7, C3, Cz, C4, T8, CP5, CP1, CP2, CP6, P7, P3, Pz, P4, P8, POz, O1, Oz, O2, and M1 and M2 [mastoids]), referenced online to CPz^33^. Both vertical and horizontal electrooculograms (EOGs) were recorded using a bipolar montage to monitor eye movements and eye-blinks. The electrode pairs were placed at the supra- and suborbit of the right eye and at the external canthi of the eyes, respectively^33^. Electrode impedance was kept below 10 kΩ. The EEG and EOG signals were amplified with Eego amplifier (ANT Neuro, Enschede, Netherlands), bandpass filtered (0.3–40 Hz), and digitized at 1000 Hz.

### Data reduction and analysis

The EEG signal was downsampled to 500 Hz and re-referenced offline to a linked mastoids montage as implemented in EEGLAB^78^. Further processing was conducted in Brainstorm^79^. The EEG was filtered offline with a band-pass filter of 0.3–30 Hz and manually corrected for blink artifacts using independent component analysis (ICA). The EEG was then segmented into 6000 epochs, from 3000 ms before stimulus onset to 3000 ms after stimulus onset, to prevent boundary effects^17^. Each epoch was baseline-corrected by subtracting the mean pre-stimulus voltage between − 250 ms and − 50 ms. Segments that contained residual artifacts exceeding ± 70 μV (peak-to-peak) were excluded. By applying the a priori criteria of excluding individuals for whom more than 50% of trials were rejected, two participants (2 females) in the group with dysphoria were excluded due to excessive noise on the EEG recording and failed mastoid, respectively. Moreover, one participant in the group with dysphoria (1 female) and one in the group without dysphoria (1 male) were excluded due to excessive noise on electrode T7 and overall low-quality signal which precluded cluster-based time–frequency analysis. On the remaining sample, the artifact rejection led to an average ± SD acceptance of 19.0 ± 3.6 pleasant trials, 18.5 ± 3.0 neutral trials, and 19.1 ± 3.2 unpleasant trials in the dysphoria group, and of 18.7 ± 3.2 pleasant trials, 18.7 ± 3.2 neutral trials, and 19.0 ± 3.1 unpleasant trials in the control group. No statistically significant differences between groups or among emotional conditions in the average acceptance of pleasant, neutral, and unpleasant trials emerged (all *p*s > .15).

Time–frequency analysis was performed using Morlet wavelet transformation on individual trials for each 1-Hz frequency bin between 1 and 30 Hz, using a mother wavelet at 1 Hz with 3-s time resolution (full width at half maximum; FWHM). Time–frequency decompositions were then averaged for each participant and emotional condition, and the event-related spectral perturbation (ERSP) was computed as the change in power expressed in decibels (dB) relative to the baseline (− 900 to − 400 ms) in each frequency bin at each time point^17^. Then, data were grand averaged across each group for each emotional condition.

### Statistical analysis

A cluster-based approach has been conducted to control over type I error rate arising from multiple comparisons across electrodes and time points^80^. This approach is advantageous as it does not rely on assumptions about the distribution of the data or the theoretical underlying distribution of test statistics under the null hypothesis (i.e., Gaussian)^81^. Instead, the distribution is generated by the data itself, by iteratively shuffling the condition labels over trials (i.e., within-subjects) or over subjects (i.e., between-subjects) and recomputing the statistics. The shuffling is repeated thousands of times until a distribution of the test statistic value observed under the null hypothesis is generated. If the observed statistic value (i.e., the test statistic associated with the non-shuffled data) falls within the distribution of the null-hypothesis test statistic values, the null hypothesis cannot be rejected and this would indicate that the observed data could have been randomly generated^81,82^. Cluster-based correction assumes that a true effect should show a temporal and spatial extension, with neighbor sensors showing similar patterns^81^. With cluster-based correction, at each iteration of the null-hypothesis distribution generation, a threshold is applied to the time–frequency map, such that the outcome is units of clusters instead of single pixels (i.e., electrodes)^81^. In the present study, once thresholded values resulted from statistics across electrodes and time points were obtained, the differences within emotional conditions or between groups were shuffled pseudo-randomly 2000 times^17,33^. To obtain a ‘null’ distribution of effect sizes, the maximal cluster-level statistics (i.e., the sum of values across contiguously significant electrodes and time points at the threshold level) were extracted for each shuffle. For each significant cluster in the (non-shuffled) data, the cluster-corrected *p*-value was computed as the statistics of the proportion of clusters in the null distribution that exceeded the one obtained for the cluster in question^17,33^. The analysis was conducted with a − 100 to 1400 ms time window and clusters with a *p*_*corr*_ < .05 were considered statistically significant.

To test within-group differences in event-related power changes among emotional categories (pleasant, neutral, unpleasant) and between-group (with dysphoria, without dysphoria) differences within each emotional category, cluster-based repeated measures ANOVAs and two-tailed unpaired *t*-tests were employed, respectively. The cluster-based statistical tests were run on the event-related delta (1–3 Hz), theta (4–7 Hz), and alpha (8–13 Hz) power over time-points in the − 100 to 1400 ms interval and a *p* < .05 criterion was employed to threshold the matrices^17^. Considering that male sex was fairly underrepresented, an independent *t*-test was carried out to determine whether sex influenced the time–frequency activity in the clusters that emerged as significant between the two groups.

## Results

### Delta power

#### Differences among emotional categories in event-related delta power

**Figure 1:**
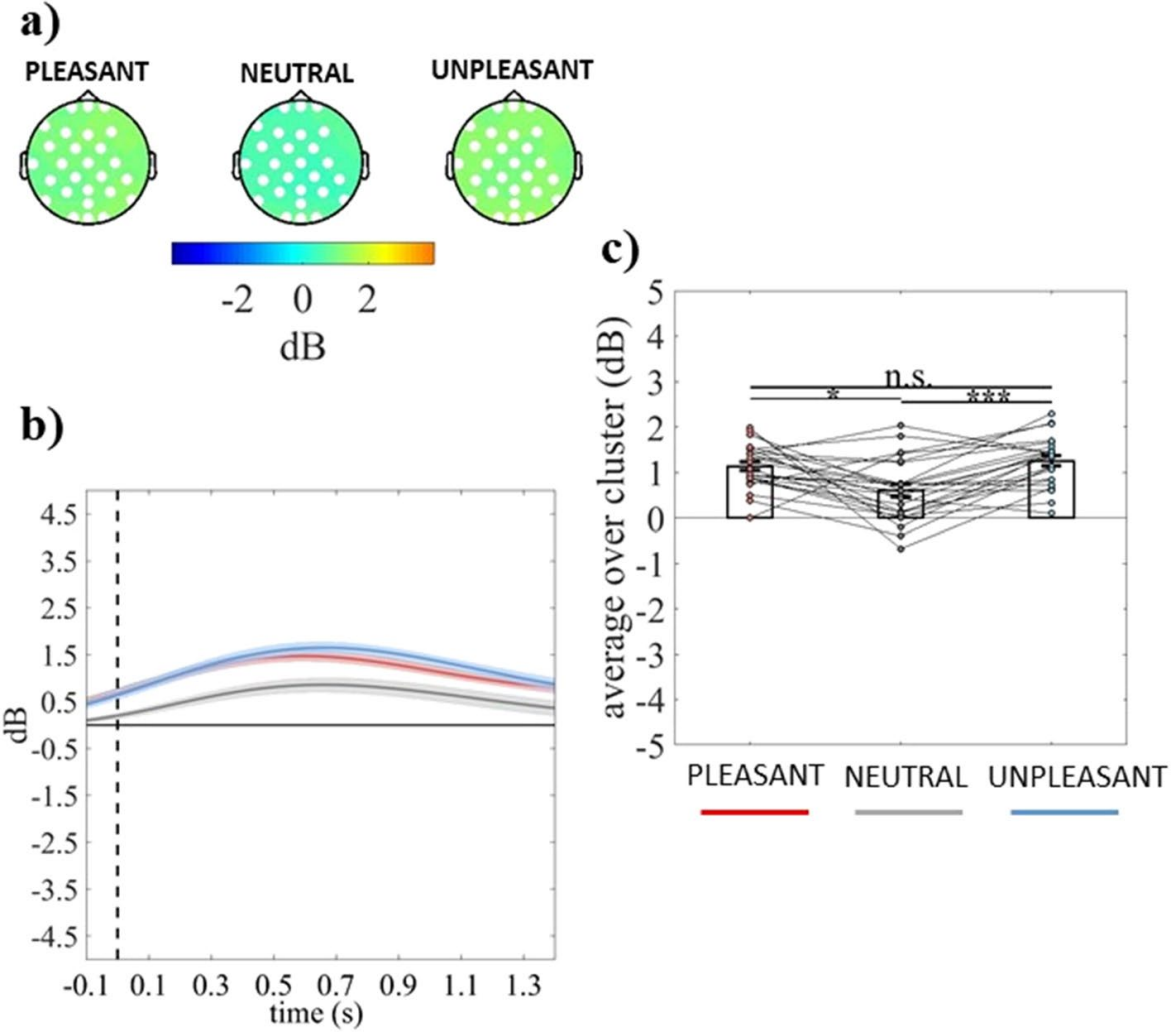
(**a**) Topography of the mean event-related delta power (dB) of individuals with dysphoria averaged over the significant time points (− 100 to 1400 ms time window) for pleasant, neutral, and unpleasant conditions. (**b**) Time course of grand-average event-related delta power of individuals with dysphoria averaged over the significant electrodes for pleasant (red line), neutral (grey line), and unpleasant (light blue line) conditions. Shaded areas represent ± standard error of the mean (SEM). (**c**) Mean event-related delta power of each participant (in the group with dysphoria) averaged over the significant electrodes and time points for pleasant, neutral, and unpleasant conditions. Each circle represents one participant; colored frames represent the mean event-related delta power across all participants and the solid black lines represent ± SEM. *p < .05; ***p < .001.

**Figure 2:**
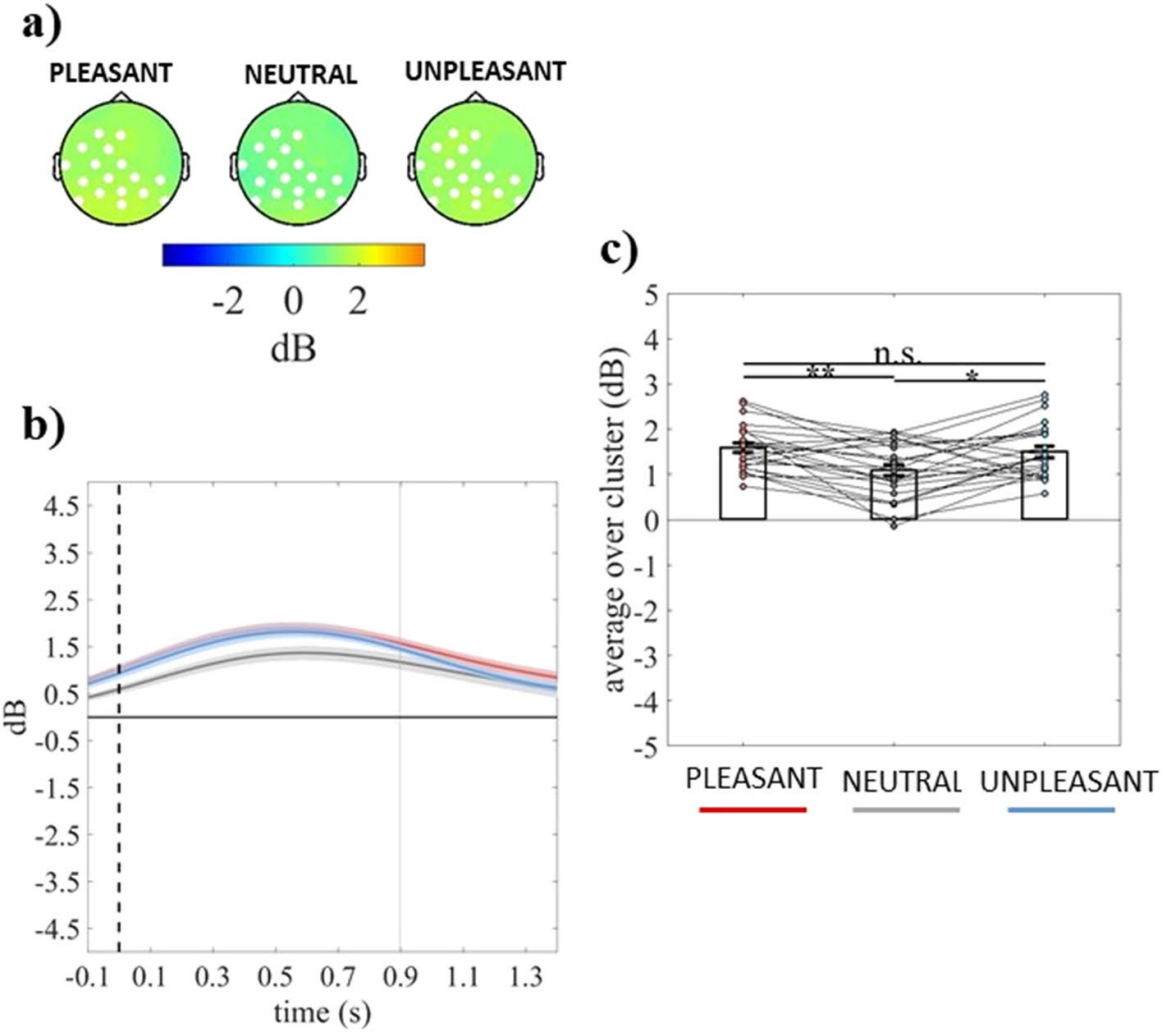
(**a**) Topography of the mean event-related delta power (dB) of individuals without dysphoria averaged over the significant time points (− 100 to 898 ms time window) for pleasant, neutral, and unpleasant conditions. (**b**) Time course of grand-average event-related delta power of individuals without dysphoria averaged over the significant electrodes for pleasant (red line), neutral (grey line), and unpleasant (light blue line) conditions. Shaded areas represent ± standard error of the mean (SEM) and the gray line represents the end of the significant time window (898 ms). (**c**) Mean event-related delta power of each participant (in the group without dysphoria) averaged over the significant electrodes and time points for pleasant, neutral, and unpleasant conditions. Each circle represents one participant; colored frames represent the mean event-related delta power across all participants and the solid black lines represent ± SEM. *p < .05; **p < .01.

The cluster-based analysis on event-related delta power showed a significant positive topographically widely distributed cluster (electrodes = FP1 FPZ FP2 F7 F3 FZ F4 FC5 FC1 FC2 T7 C3 CZ C4 CP5 CP1 CP2 CP6 P7 P3 PZ P4 P8 POZ O1 OZ O2) in the group with dysphoria (cluster *F*-value_max_ = 278,357.09, *p*_*corr*_ = .001, time window = − 100 to 1400 ms) and a significant positive topographically widely distributed cluster (electrodes = F3 FZ FC5 FC1 T7 C3 CZ CP5 CP1 CP2 CP6 P7 P3 PZ P4 P8 POZ) in the group without dysphoria (cluster *F*-value_max_ = 67,817.30, *p*_*co*rr_ = .01, time window = − 100 to 898 ms), as shown in panel a of Figs. 1 and 2, respectively. Both groups showed significantly greater delta to pleasant and unpleasant stimuli relative to neutral ones (all *p*s ≤ .017; Figs. 1 and 2b,c).

#### Differences between groups in event-related delta power for each emotional category

**Figure 3:**
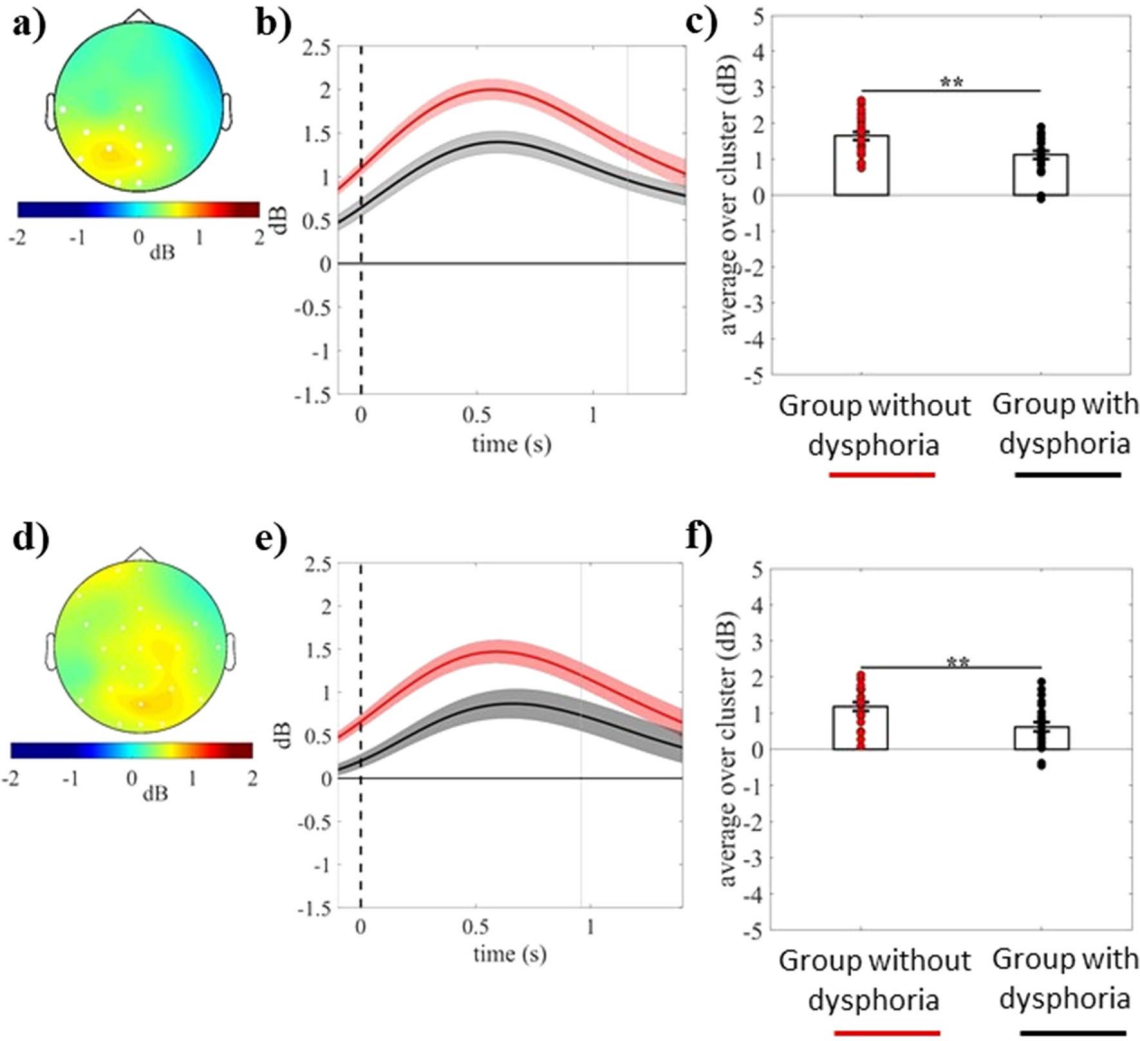
(**a**,**d**) Topography of the mean difference between groups in event-related delta power (dB; group without dysphoria minus group with dysphoria) averaged over the significant time points (**a**; − 100 to1148 ms time window) for the pleasant and (**d**; − 100 to 958 ms time window) neutral condition, respectively. (**b**) Time course of grand-average event-related delta power averaged over the significant electrodes for pleasant and neutral conditions in the group with dysphoria (black line) and the group without dysphoria (red line). Shaded areas represent ± standard error of the mean (SEM); the gray line represents the end of significant time windows. (**c**) Mean event-related delta power of each participant in the group with dysphoria and the group without dysphoria averaged over the significant electrodes and time points for the pleasant condition and (**f**) neutral condition. Each circle represents one participant; the frames represent the mean event-related delta power across all participants in the group with dysphoria and the group without dysphoria and the solid black lines represent ± SEM. **p < .01.

Cluster-based unpaired *t*-tests on event-related delta power revealed significant positive clusters for the difference between the two groups within pleasant (electrodes = T7 CZ CP5 CP1 P7 P3 PZ P4 POZ O1 OZ; cluster *t*-value_max_ = 14,504.83, *p*_*corr*_ = .03, time window = − 100 to 1148 ms) and neutral (electrodes = FP1 FPZ F7 FZ FC5 FC1 FC2 FC6 C3 CZ C4 T8 CP1 CP2 CP6 P7 P3 PZ P4 P8 POZ O1 OZ O2; cluster *t*-value_max_ = 24,222.04, *p*_*corr*_ = .02, time window = − 100 to 958 ms) conditions, as shown in panels a and d of Fig. 3. Specifically, the dysphoria group showed reduced delta in response to both pleasant and neutral stimuli relative to the group without dysphoria (see Fig. 3, panels b, c, e, and f). Unpaired *t*-test did not reveal any significant cluster for the difference between the groups within the unpleasant condition. Moreover, delta power within the significant clusters that emerged from the between-groups comparisons was not influenced by sex (neutral cluster, *p* = .125; pleasant cluster, *p* = .270).

### Theta power

#### Differences among emotional categories in event-related theta power

**Figure 4:**
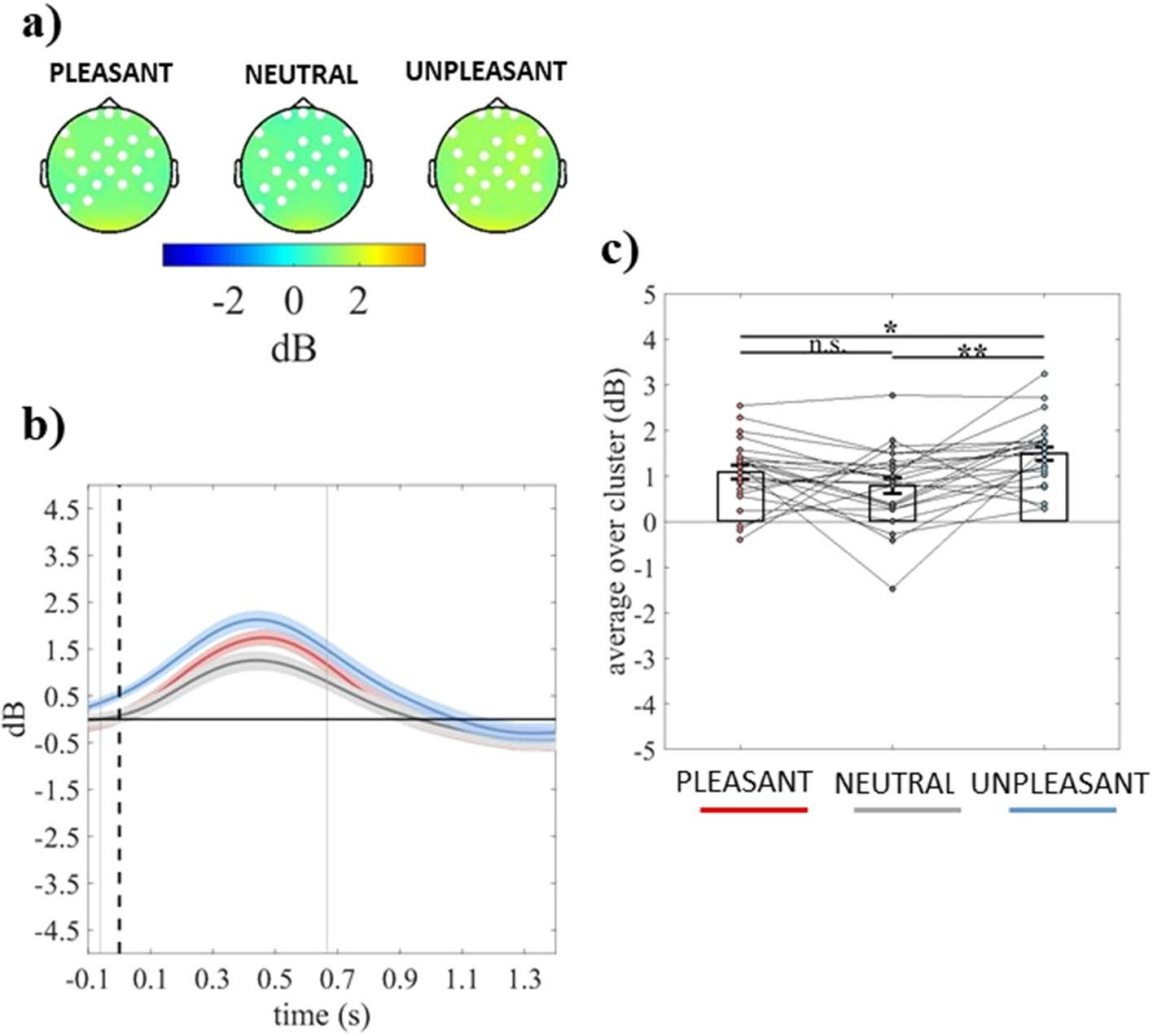
(**a**) Topography of the mean event-related theta power (dB) of individuals with dysphoria averaged over the significant time points (− 60 to 666 ms time window) for pleasant, neutral, and unpleasant conditions. (**b**) Time course of grand-average event-related theta power of individuals with dysphoria averaged over the significant electrodes for pleasant (red line), neutral (grey line), and unpleasant (light blue line) conditions. Shaded areas represent ± standard error of the mean (SEM) and gray lines represent the significant time window (− 60 to 666 ms). (**c**) Mean event-related theta power of each participant (in the group with dysphoria) averaged over the significant electrodes and time points for pleasant, neutral, and unpleasant conditions. Each circle represents one participant; colored frames represent the mean event-related theta power across all participants and the solid black lines represent ± SEM. *p < .05; **p < .01.

**Figure 5:**
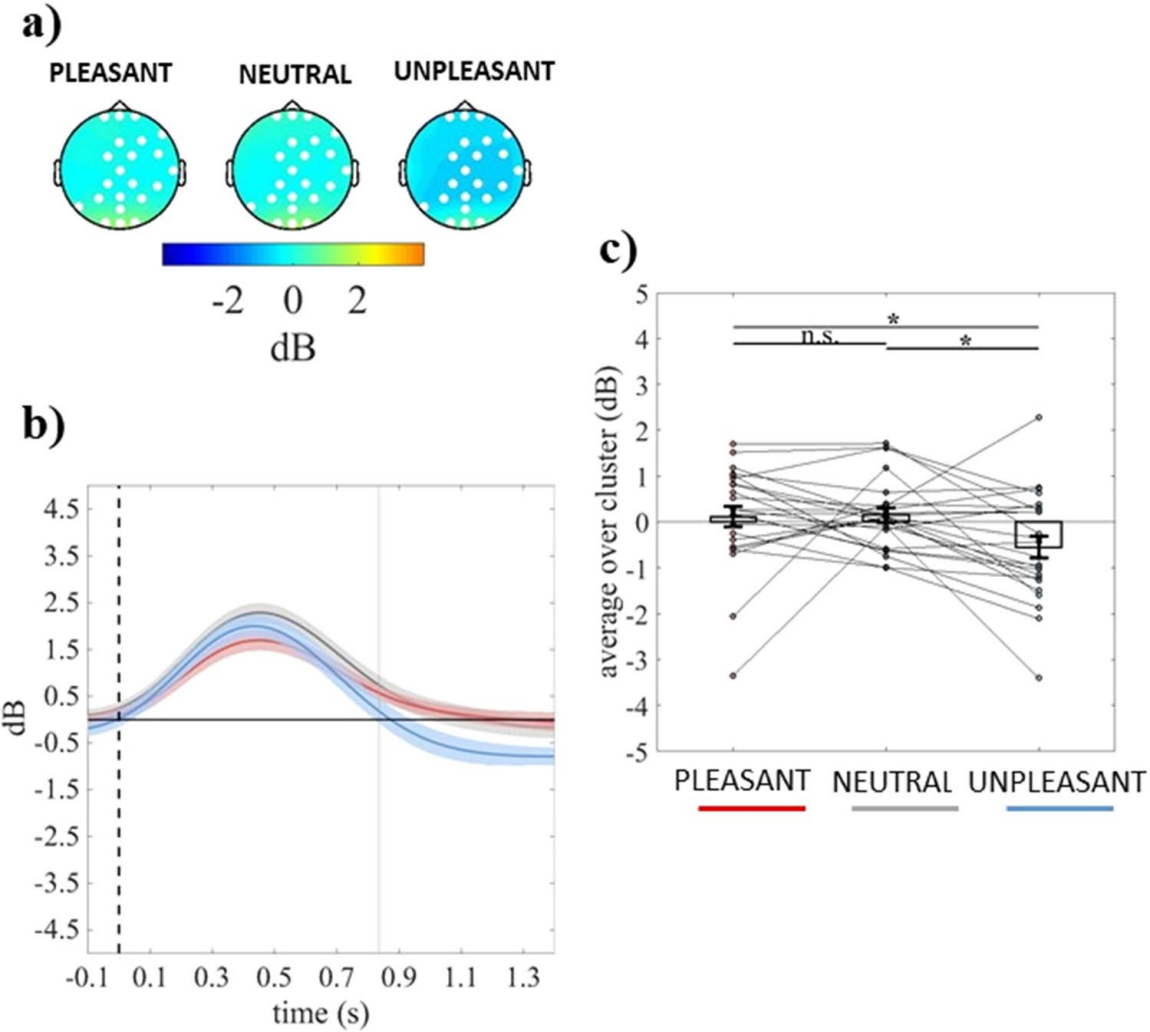
(**a**) Topography of the mean event-related theta power (dB) of individuals without dysphoria averaged over the significant time points (836 to 1400 ms time window) for pleasant, neutral, and unpleasant conditions. (**b**) Time course of grand-average event-related theta power of individuals without dysphoria averaged over the significant electrodes for pleasant (red line), neutral (grey line), and unpleasant (light blue line) conditions. Shaded areas represent ± standard error of the mean (SEM) and the gray line represents the beginning of the significant time window (836 ms). (**c**) Mean event-related theta power of each participant (in the group without dysphoria) averaged over the significant electrodes and time points for pleasant, neutral, and unpleasant conditions. Each circle represents one participant; colored frames represent the mean event-related theta power across all participants and the solid black lines represent ± SEM. *p < .05.

The cluster-based analysis on event-related theta power showed a significant positive fronto-centro-parietal cluster (electrodes = FP1 FPZ FP2 F7 FZ F4 F8 FC5 FC1 FC2 FC6 C3 CZ C4 CP5 CP1 CP2 CP6 P7 P3) in the group with dysphoria (cluster *F*-value_max_ = 41,826.52, *p*_*corr*_ = .03, time window = − 60 to 666 ms) and a significant positive fronto-centro-parieto-occipital cluster (electrodes = FP1 FPZ FP2 FZ F4 F8 FC1 FC2 FC6 CZ T8 CP1 CP2 CP6 P7 P3 PZ P4 POZ O1 OZ O2) in the group without dysphoria (cluster *F*-value_max_ = 39,465.97, *p*_*corr*_ = .03, time window = 836 to 1400 ms), as shown in panel a of Figs. 4 and 5, respectively. The group with dysphoria showed increased theta power in response to unpleasant than neutral and pleasant stimuli (all *ps* ≤ .022; Fig. 4b,c). Differently, the group without dysphoria revealed reduced theta power in response to unpleasant than neutral and pleasant stimuli (all *ps* ≤ .016; Fig. 5b,c).

#### Differences between groups in event-related theta power for each emotional category

Unpaired *t*-test conducted on event-related theta power did not reveal any significant cluster for the difference between the groups within each emotional condition (all *p*s ≥ .125).

### Alpha power

#### Differences among emotional categories in event-related alpha power

The cluster-based analyses on event-related alpha power did not reveal any statistically significant cluster in testing possible within-group differences (all *p*s ≥ .088).

#### Differences between groups in event-related alpha power for each emotional category

Unpaired *t*-test conducted on event-related alpha power did not reveal any significant cluster for the difference between the groups within each emotional condition (no cluster was detected; hence *p*-values were not generated).

## Discussion

The present study examined affective disposition and cognitive processing in dysphoria through the analysis of the time–frequency changes within delta, theta, and alpha bands during the exposure to emotional pictures^72^. Regarding affective disposition, the dysphoria group was expected to show a hypoactivation of the approach-related motivational system and, as suggested by the ECI model, a hypoactivation of the withdrawal-related motivational system. Second, the dysphoria group was expected to show selective facilitated top-down processing of unpleasant and a reduced processing of pleasant pictures.

With respect to affective disposition, a pattern of increased event-related delta in response to all affective relative to neutral pictures emerged within both groups, indicating an affective modulation regardless of valence. Moreover, in line with the hypothesis, individuals with dysphoria showed an extended reduction of delta to pleasant pictures relative to the group without dysphoria. This finding possibly indicates a reduced emotional responding to pleasant images and a hypoactivation of the approach-related motivational system in individuals with dysphoria. Indeed, delta oscillations are linked to motivational processing, whereby an increase in its power indicates the identification of potentially rewarding cues (e.g.^42,46,49,62^). Hence, reduced delta to pleasant images could denote reduced emotional responding to pleasant/rewarding stimuli in dysphoria. Overall, considering that reduced P3/LPP complex to pleasant images was observed in dysphoria^33^, two interrelated processes seem to characterize dysphoria: a hypoactivation of the approach-related motivation system and reduced motivated attention to positively valenced content. Additionally, the dysphoria group showed reduced delta to neutral pictures than the group without dysphoria, a pattern probably due to participants’ motivational inertia, characteristic of depressive symptoms. Namely, it is plausible that the decreased motivation in dysphoria extended to non-relevant stimuli that did not elicit a saliency-detection process as prominent as in controls. Taken together, these findings provide support for the positive attenuation hypothesis in dysphoria. Conversely, the present findings are at odds with the negative potentiation hypothesis as well as with the reduced reactivity to unpleasant stimuli^20^. However, blunted reactivity to unpleasant stimuli might specifically be a manifestation of clinical depression.

Furthermore, no significant difference between the dysphoria and the control group was found in the event-related alpha. This null finding may be due to different methodological approaches employed across studies. For instance, the present study differs in several methodological features from the few previous studies that employed a time–frequency approach^18,57^. Particularly, compared to a previous study^18^, here an even more rigorous statistical approach was employed, whereby group level analyses were conducted on distinct time windows identified through cluster-based analysis conducted within each group separately. Also, although reduced alpha desynchronization to pleasant stimuli was reported in depression, results are still inconsistent in the literature (for a review see^54^).

Regarding affective cognitive processing, the two groups showed distinct patterns of theta power changes at the within-subjects level. Of note, these within-subjects differences occurred at distinct time windows, indicating potentially different processes occurring within the same stage of stimulus analysis. In the literature, two stages of emotional processing of theta power were identified: an early increase (~ 300 ms) related to automatic orienting and a later (after 300 ms) increase related to fine-grained top-down processing of salient stimuli^42,50^. Regarding the differences within the dysphoria group, an early increased in theta power for unpleasant pictures lasted until a later processing stage. On the other hand, within the control group, reduced theta for unpleasant pictures was evident only during a subsequent processing stage (836–1400 ms). It could be hypothesized that during the early stage of processing, individuals with dysphoria showed a preferential early orienting for unpleasant relative to both pleasant and neutral images. However, this effect was stable even after the early orienting stage, indicating that individuals with dysphoria performed a selective top-down processing towards unpleasant cues. Also, this pattern suggests that dysphoria may show a reduction in orienting towards pleasant pictures, which are processed as neutral ones. In contrast, controls showed a late reduction of top-down processing for unpleasant cues, suggestive for a conscious and adaptive regulation of these stimuli^83^. Consistently, a previous study on healthy participants reported that a late (1000–4000 ms) theta activity decrease was associated with reappraisal, a regulation strategy aimed at modifying the meaning of an emotional situation^83^. The late dampening of theta by reappraisal was interpreted as decreased prioritization of the stimuli by selective attention, following an initial evaluation of their affective saliency^83^. Hence, the within-subjects pattern in dysphoria not only is consistent with a facilitated processing of unpleasant cues, but it might indicate a lack of adaptive regulation strategies as compared to controls. Since theta band has been largely associated with high-order cognitive processes^84,85^, in future studies it would be interesting to investigate event-related theta while participants engage in complex affective cognitive task.

From the current findings, the distinct role of delta and theta is supported. For instance, albeit speculative, not only they represent distinct functional correlates of affective processing, but they seem to be distinctively associated with the elaboration of pleasant and unpleasant content, respectively. In this regard, previous time–frequency studies on reward and loss processing have linked increased delta to reward sensitivity and increased theta to loss processing, describing them as two dissociable processes (e.g.^86,87^). Interestingly, a previous study reported that depressive symptoms were prospectively predicted by diminished reward-related delta but not loss-related theta^87^. Despite these studies employed a different paradigm, the present findings support the perspective of a pleasantness-related delta band and unpleasantness-related theta band. The current findings on theta are novel and future studies are warranted to better disentangle its role in the top-down processing of affective stimuli in dysphoria.

The time–frequency approach applied in the present study offers several methodological advantages compared to standard ERPs. Indeed, this approach allowed the separation of two peculiar measures of affective elaboration reflecting distinct processes occurring simultaneously, namely affective disposition and top-down processing. Furthermore, in addition to the analysis of evoked oscillations, time–frequency analysis also incorporates induced oscillations, known to carry important information about cognitive processes^40^.

Some limitations should be acknowledged. First, considering that the present study was based on a community sample and that dysphoria is more prevalent among the female population^3^, most of the participants within the dysphoria group belonged to the female sex. This sex unbalance might not allow the generalization of the findings to the male population and future studies are warranted to replicate the findings and increase their generalizability. Furthermore, although the emotional passive viewing task is a valid and widely used paradigm to study affective processing (e.g.^19,38^), future studies that include specific experimental manipulations during the exposure to emotional stimuli are warranted to clarify the functional correlates of delta, theta and alpha frequency bands in affective tasks.

In conclusion, the present time–frequency study granted novel evidence on distinct but interrelated facets of affective elaboration in dysphoria, mainly characterized by a hypoactivation of the approach-related motivational system and a sustained facilitated cognitive processing along with reduced adaptive regulation of unpleasant stimuli. Considering that dysphoria is a condition known to considerably increase the risk of depression, these patterns of affective processing may represent quantitative measures allowing for early identification and treatment of depressed mood.

## Supplementary Information

Supplementary Information.

## Data Availability

The datasets analyzed during the current study are not publicly available due ethical concerns but are available from the corresponding author on reasonable request.
