# Supplementary material for: EEG time–frequency analysis reveals blunted tendency to approach and increased processing of unpleasant stimuli in dysphoria
Source: Sci Rep. 2022 May 17;12:8161. doi: 10.1038/s41598-022-12263-9 (PMC9113991; doi:10.1038/s41598-022-12263-9)
Supplement: Supplementary file 1 — Supplementary Information. [file 41598_2022_12263_MOESM1_ESM.pdf]

Supplementary material

The selected IAPS pictures were the following: 1050, 1114, 1120, 1300, 1302, 1930, 1932, 3500, 4611, 4647, 4651, 4652, 4660, 4664, 4670, 4680, 4683, 4690, 4695, 4810, 6200, 6210, 6230, 6242, 6243, 6244, 6250, 6260, 6312, 6313, 6370, 6510, 6540, 6550, 6560, 7000, 7002, 7004, 7009, 7010, 7020, 7035, 7036, 7041, 7050, 7056, 7059, 7130, 7175, 7224, 7233, 7242, 7491, 7500, 7547, 7560, 7595, 7700, 7950, 8030, 8031, 8034, 8080, 8161, 8180, 8185, 8186, 8200, 8370, 8400, 8490, 9425.
